# Development of a Smart Health Care Service Using Metaverse and Chatbot Technologies for Adolescents, Parents, and School Health Teachers: User-Centered Design Approach

**DOI:** 10.2196/69190

**Published:** 2025-07-03

**Authors:** Hana Kim, Jae-Heon Kang, Yeeun Kwon, Che Eun Kim, Jung han Yu, Hyo Seung Nam, Jisan Lee

**Affiliations:** 1 Department of Nursing Hoseo University Asan Republic of Korea; 2 Department of Family Medicine Sungkyunkwan university Kangbuk Samsung Hospital Seoul Republic of Korea; 3 Department of Healthcare Planning Kangbuk Samsung Hospital Seoul Republic of Korea; 4 Application Development MARBLELOTUS Inc Seoul Republic of Korea; 5 Department of Nursing Gangneung–Wonju National University Gangneung Republic of Korea

**Keywords:** adolescent, exercise, needs assessment, mobile health, IT

## Abstract

**Background:**

Adolescent health issues, particularly obesity, have become increasingly serious, highlighting the need for health management strategies tailored to the unique life cycle characteristics of adolescents.

**Objective:**

This study aimed to develop a smart health care service for adolescents, their parents, and school health teachers to enhance their health.

**Methods:**

The service leverages a mobile app, a web platform, wearable devices, the metaverse, and chatbots. The development process included a needs assessment, core user interface (UI) design, and service model creation. The needs assessment involved a literature review of 65 studies, a web survey of 96 participants, and 30 interviews. A usability evaluation of the core UI, shaped by the insights from the needs assessment, was conducted with 76 participants.

**Results:**

The service was conceptualized emphasizing school settings to cater to school-aged users, ensuring that the design is closely aligned with user needs. In addition, it incorporates features to deliver personalized services based on individual health information and uses gamification elements to enhance user engagement. The core UI was developed in response to the needs assessment findings, supported by a user flowchart. Furthermore, we created a use case diagram illustrating the interaction between various users and the services, a flowchart outlining the service algorithm, and a lifelog data collection system. The core UI usability evaluation results revealed that both students and parents considered sleep management important, while school health teachers deemed the measurement of physical activity essential. Students and parents prioritized physical activity measurement, and students particularly favored rewards for activities as the most promising solutions for health management challenges. The outcomes of the core UI usability evaluation indicated that school health teachers rated effectiveness (mean 4.26, SD 0.41), usefulness (mean 4.18, SD 0.54), usability (mean 4.29, SD 0.55), and user control (mean 4.08, SD 0.60) highly, reflecting the highest expectations across all categories.

**Conclusions:**

This study demonstrated that a school-based smart health care service can effectively support adolescent health management by integrating personalized health data, gamification, and interactive tools. The usability evaluation revealed that students and parents prioritized sleep and physical activity tracking, while school health teachers emphasized the importance of monitoring physical activity. In addition, reward-based engagement strategies were identified as a promising approach to improve adolescent health behaviors. These findings suggest that leveraging digital health solutions tailored to adolescents’ needs can contribute to establishing sustainable health habits from an early age.

## Introduction

### Background

The World Health Organization predicts that 1 in 8 children and adolescents aged 5 to 19 years will be obese by 2030, highlighting childhood and adolescent obesity as the most pressing public health issue of the 21st century [1]. Recent data show a substantial increase in the prevalence of obesity among Korean adolescents, rising from 15.1% in 2019 to 19% in 2021 [2]. During adolescence, individuals gain more autonomy compared to childhood; however, despite being aware of healthy behaviors, they often struggle to implement them [3]. In addition, adolescence is a time when physical activity levels decline sharply, with studies indicating that 81% of adolescents aged 11 to 17 years worldwide are physically inactive [4]. Teenagers have more freedom to choose their meals outside the home, so they are more likely to opt for fast food and prioritize taste over nutrition [5]. Transitioning from childhood to adulthood can be stressful as they navigate various physical, emotional, and social changes [6]. Hormonal shifts during puberty also influence appetite and weight, increasing the risk of emotional eating [5]. The typical lifestyle of adolescents tends to involve physical inactivity, sedentary behavior, and poor dietary habits, which can contribute to obesity, a decline in cardiopulmonary fitness, increased body fat, and a heightened risk of cardiovascular disease [7]. Implementing preventive health services during adolescence can help modify or mitigate health risk behaviors, promote the development of healthy habits, and enhance overall well-being [8].

Health care services aimed at adolescents need to focus on strategies that align with their unique physical and cognitive developmental characteristics [9]. Adolescence is a period marked by significant physical, mental, and social changes, resulting in distinct health and developmental needs that differ from those in other life stages [10]. Furthermore, adolescents are less likely to engage with services they perceive as unsuitable, making it crucial for health care offerings to reflect their specific requirements [11]. Recent surveys indicate that users prefer personalized services tailored to their individual health status and needs [12]. The absence of personalization in digital services has been identified as a factor contributing to increased dropout rates, implying that the effectiveness of these services may be diminished without customized support [13].

School-based health screenings promoting student health have shown limited evidence regarding their effectiveness, feasibility, and acceptability [14]. Furthermore, some schools have indicated that they do not use the results of these health screenings for case management [15]. In South Korea, comprehensive health screenings are conducted in elementary, middle, and high schools under the School Health Act; however, school health programs primarily emphasize physical activity, resulting in limitations in addressing various health areas [16]. In addition, the continuous operation of health programs faces challenges due to a shortage of medical personnel, hindering comprehensive health services [17].

This study seeks to develop a digital intervention aimed at promoting health among adolescents. Research has shown that digital interventions for adolescents can yield various benefits, including enhancing body image, improving mental health, preventing substance abuse, and encouraging physical activity [18]. Mobile apps, in particular, are recognized as practical intervention tools due to their widespread use, convenience, and cost-effectiveness. These apps can be crucial in monitoring and managing health behaviors, helping adolescents cultivate healthy lifestyle habits through daily tracking [19]. Moreover, a recent survey indicates that 95% of adolescents aged 13 to 17 years use smartphones and are online nearly all the time or several times a day [20]. This suggests that a digital intervention incorporating a mobile app will provide an easily accessible service for adolescents. In addition, the school-centered approach aims to increase the acceptability and sustainability of the program and to include parents and school nurses in the service to increase their sense of ownership of the program, which will help schools adapt the program to their needs [21]. In addition, the school-centered approach can help address equity issues due to the digital divide [22]. Parental participation is essential in creating a healthy environment at home and can play a positive role in adolescent health [23].

### Objectives

This study aims to design a smart health management service using the web, wearable devices, the metaverse, and chatbots centered around a mobile app for effective adolescent health management, with schools, parents, and health teachers participating.

## Methods

### Study Design

**Figure 1:**
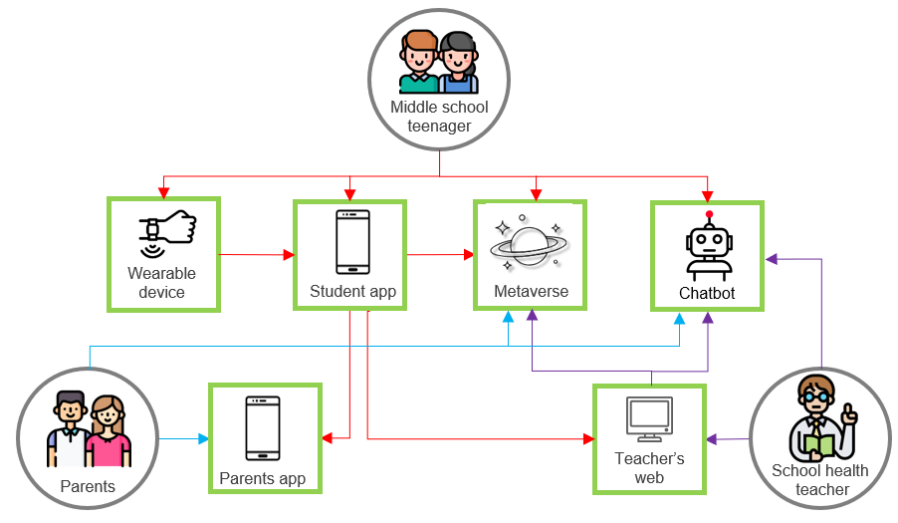
Smart health care service composition for adolescents.

This study aims to design a smart health care service called MUZZIM. “MUZZIM” is a slang term that means “cool” in Korean, and it was chosen as the service name to make it appealing to adolescents. The service uses a mobile app, web integration, wearable devices, a metaverse platform, and chatbots (Figure 1). It is tailored for adolescents, their parents, and school health teachers to enhance adolescent health.

### Study Process

This study aims to assess the needs and design the core user interface (UI) design for a smart health care service tailored for adolescents, ultimately developing the MUZZIM service model based on this study.

### Needs Assessment and Core UI Design for Smart Health Care Service

#### Literature Review

A literature review was conducted to identify the characteristics of previous studies targeting children and adolescents. In addition, even if it was not a study targeting children or adolescents, studies that included content that could be used as a reference in this service model were included in the list of reviewed literature through agreement among researchers. This outlines the primary service areas intended for adolescents, identifies the fields for expert counseling, and describes the specific methods of service operation.

#### Smart Health Care Service Needs Assessment and Core UI Design

In the initial phase of designing a smart health care service, a needs assessment was conducted, drawing on a previous study highlighting the importance of incorporating the characteristics and preferences of adolescents in digital health solutions [9]. The needs assessment was conducted by conducting a survey and interviews with middle school students who are service users and their parents and school health teacher. The survey aimed to clarify the needs for the service by using the “user needs for service areas, expert consultation areas, and digital service methods” derived from the literature review results. Furthermore, we assessed digital health readiness—specifically mobile health (mHealth) literacy and equity—and self-efficacy in using digital media systems. This evaluation aimed to ensure the delivery of services tailored to the target participants and to enhance health equity. During the interview process, we used the Method for Deriving Users’ Needs, which is part of the Method of App Selection based on User Needs (MASUN) framework [24-26]. MASUN is a methodology designed to assist health care service providers, researchers, and users in selecting the most suitable mHealth apps [26]. Among them, the Method for Deriving Users’ Needs is a process in which the target group that will use the service directly identifies the needs of the relevant group through brainstorming, mind mapping, writing personas and scenarios, deriving a needs list, and designing a core UI that reflects this [26]. In this study, the research team recognized that the interview participants might not fully grasp all the representative health issues affecting adolescents. Therefore, expert insights were sought to highlight recent prevalent health concerns in this age group. On the basis of this information, personas and scenarios were crafted according to the priorities of each health area to guide the interviews. In addition, the development of the core UI was substituted with a preexisting example of a child-focused app created by the researcher, allowing for the collection of feedback on the UI.

The questionnaire comprised 47 questions. This included 6 general characteristics: participant classification, age (of parents and health teachers), gender, occupation, willingness to engage with smart health care services (among students and parents), and the perceived usefulness of wearable devices in smart health care apps. In addition, the questionnaire featured 33 items focused on digital readiness, which encompassed mHealth literacy and equity, along with 4 questions related to self-efficacy in using digital media systems. The digital readiness items were derived from the tool developed by Kim et al [27]. The self-efficacy measurement tool for using digital media systems was based on research by Hong et al [28], with subsequent translation and modification by Kim [29] into Korean, which the researcher further adapted for this study. Furthermore, the 4 questions crafted by the research team aimed to identify the demand for health management services, covering topics such as desired service areas for smart health care, areas for expert consultation and evaluation, types of content, and preferred digital devices.

The interview used a semistructured format using questionnaire items. After drafting these items, a pilot test was conducted with 2 middle school students to assess their understanding and engagement. The research team then reviewed, revised, and supplemented the questions accordingly. On the basis of the survey and interview findings, a list of user needs for the service was created, leading to the development of a core UI designed to address these needs. The core UI design for the app and website was finalized after the research team drafted the initial concepts and a designer refined them. Microsoft PowerPoint 2021 was used for the core UI design.

#### Usability Evaluation of Smart Health Care Service Core UI

At this stage, a survey was conducted to evaluate the usability and prioritize the core UI elements for the smart health care service. This core UI was developed based on the needs identified in the needs assessment. The survey comprised 50 questions: 6 questions regarding the importance of UI items, 6 questions on the potential for problem resolution, 6 questions assessing user preference, 12 questions on the heuristic evaluation of mobile apps, and 20 questions focused on evaluating the usability of the core UI. Notably, the 12 heuristic evaluation questions presented some challenges for adolescents, so they were administered only to participants who expressed interest. The 18 questions evaluating the importance, potential for problem-solving, and preference for UI items were crafted by the research team; each item was rated on a scale from 1 to 4, with higher scores indicating greater importance, problem-solving capability, and user preference. The heuristic evaluation of mobile apps used the framework established by Joyce et al [30], with scores ranging from 1 to 3 points, where a higher score indicated more usability challenges. This tool also collected a variety of user feedback, including additional suggestions. The usability of the core UI was evaluated using the Korean Health Information Technology Usability Evaluation Scale [24], which uses a 5-point scale, with higher scores indicating greater usability.

### Development of MUZZIM

#### Development of User Flowchart for Apps and Web to Provide MUZZIM Service

In response to the requirements for adolescent health care services and the findings from usability evaluations of the core UI, enhancements were implemented in both the app and web versions. A user flowchart was also created using Microsoft PowerPoint 2021 to illustrate the specific use paths for service users.

#### Algorithm Development of MUZZIM and Design of the Lifelog Data Collection System

A use case diagram was developed to effectively illustrate the interactions between the components of this service, such as the mobile app, web, wearable devices, metaverse platforms, and chatbots. In addition, a flowchart was created to visualize the underlying algorithm. To determine the most suitable wearable device (specifically a smartwatch) for this service, we reviewed information from existing health care apps and wearable devices, resulting in a list of proposed function implementations. In addition, we analyzed the data format, collection method, and device used for gathering the lifelog data in this study, and a diagram illustrating the lifelog data collection path was created. The creation process used Microsoft PowerPoint 2021.

### Study Population and Sampling

The study participants were first-year middle school students identified as having a high demand for health management services among adolescents. In addition, the study included parents of these students and middle school health teachers who could support adolescents in using the service. Recruitment of students and parents took place at 4 middle schools in Seoul, South Korea. Students and parents interested in participating were enlisted after distributing a recruitment notice and holding an information session for each school. The school health teachers were recruited by emailing the recruitment notice from the Seoul Metropolitan Office of Education, instructing them to contact the researcher if they wished to participate. The number of participants for the survey and interview was determined based on previous studies [24-26,31] that implemented the same service development methodology used in this study. A total of 90 individuals participated in the survey (30 students, 30 parents, and 30 health teachers), and 30 individuals participated in the interview (10 students, 30 parents, and 30 health teachers). Participants completed a needs assessment survey and a usability evaluation questionnaire. However, multiple researchers engaged in simultaneous recruitment during the recruitment phase, including 6 additional students. The survey was administered twice, and the first health care service needs assessment survey included responses from 96 participants (36 students, 30 parents, and 30 school health teachers) in the final analysis. In the second survey, the usability evaluation of the needs items and core UI was conducted. The questionnaire was distributed to the same 96 participants as the first survey, and 76 copies were returned and included in the analysis. Among the student respondents who participated in the usability evaluation, 16 chose to engage in a heuristic review conducted solely for those interested.

### Data Collection

The recruitment of research participants took place from November 23 to 30, 2022, through a notice aimed at potential participants. To facilitate participant recruitment, the researcher sought the cooperation of the Seoul Metropolitan Office of Education, ultimately selecting 4 schools where recruitment notices were distributed. Individuals involved in the study completed 2 web surveys (needs assessment and usability evaluation). In addition, 10 applicants from each group were interviewed to assess their demand for smart health care services. Before the surveys and interviews, a pilot test was conducted with 2 middle school students to refine the questions.

### Ethical Considerations

Approval from the Bioethics Committee’s institutional review board was secured before proceeding with the study (KBSMC2022-10-061-002). The researcher personally visited these schools to explain the study’s purpose, the voluntary nature of participation, confidentiality, and other relevant details to interested participants. Written consent was obtained, and a mobile survey link was sent to their devices. For middle school students, the survey was conducted only after obtaining consent from their guardians, who also signed a written consent form. Participants who completed the study received a small incentive: a mobile gift certificate. Participants who completed the study were given a mobile gift certificate worth KRW 10,000 (US $7.2) if they participated in the survey and KRW 100,000 (US $72,4) if they participated in the interview. The data collected through the study will be used only for the study and will be anonymized after collection. Electronic data will be stored on a password-protected computer accessible only to the researcher. In addition, the collected data will be stored for up to 3 years after the end of the study, after which the written data will be shredded, and the electronic data will be permanently deleted.

### Data Analysis

The collected data were analyzed using SPSS for Windows (version 29.0; IBM Corp). Descriptive statistics were used to explore participants’ general characteristics and main variables.

## Results

### Needs Assessment and Core UI Design for Smart Health Care Service

#### Literature Review

We conducted a review of 65 studies and reports examining the provision of health care services—encompassing physical activity, diet, and mental health—for children and adolescents through information and communication technology (ICT), both in South Korea and abroad. Our literature review also included relevant studies that may pertain to other age groups. Of these 65 studies, 24 (37%) specifically addressed physical activity. Our analysis of 34 widely used domestic physical activity apps showed that most apps offered training coaching and voice training coaching [32]. Furthermore, features that allowed users to share their records and community content were the most commonly used strategies to encourage ongoing app engagement [32]. An analysis of 30 domestic and international web-based physical activity promotion services highlighted several key content areas: measurement (including health and physical fitness information), evaluation (individual evaluation reports based on measurements), prescription (the setting of individual health and fitness goals and guidelines), use (provision of physical activity promotion programs and educational materials), and management (ensuring service convenience for users) [33]. To enhance web-based physical activity promotion services, several strategies were identified: offering feedback to users, providing integrated content, incorporating enjoyable elements, fostering motivation, linking with local communities and related organizations, creating environments conducive to physical activity, and generating revenue for private companies [33]. The primary forms of ICT used for physical activity interventions included apps, wearable devices (such as smart bands), web platforms, SMS text messaging, smartphones, and exergames. A study incorporating game elements in physical activity interventions confirmed an increase in activity levels and demonstrated improvements in objective health indicators, including BMI, abdominal subcutaneous fat, and blood pressure [34]. This indicated that integrating game elements was effective for the targeted age group in these interventions.

A total of 16 studies were identified for improving nutritional status. A user needs analysis—targeting children and adolescents, parents, and health care managers—conducted to develop a child and adolescent obesity management platform revealed that users expressed a need for a health management app that supports joint participation by children and adolescents and their parents, allows for recording exercise or meal diaries with monitoring by health care managers, enables activity target setting based on calories burned, provides customized exercise programs and diets, offers automatic expert evaluation of exercise and meal information, and integrates an obesity management platform based on an activity tracker [35]. In the study by Kin and Lee [36], there was a recognized need for content with simple and organized text and concise graphics using characters; content differentiated by sex and age; and a counseling site that was easy, detailed, friendly, and provided answers quickly. The primary forms of ICT used included smartphones, apps, SMS text messaging, web, and email, and the management programs were often implemented for overweight and obese adolescents. When comparing the effects on normal-weight adolescents and overweight adolescents, they were found to be more effective for overweight or obese adolescents. Motivations for using diet management apps differed by sex. Female students were primarily motivated by positive feedback, recognition, and enjoyment, while male students were more influenced by reward systems and notification features. It was found to be less effective when relying on self-monitoring, and it was found to be significantly effective when game elements were included. In South Korea, numerous diet management programs have been implemented by local governments; however, these initiatives primarily target adults, and it has been challenging to identify programs that specifically address adolescent populations and provide measurable outcomes.

A total of 20 studies were identified concerning the promotion of mental health. A survey conducted on mobile health preferences among individuals aged 5 to 25 years revealed that most respondents (68.2%) favored smartphone apps over web-based options; in addition, 53.9% of the respondents desired apps that include social networking or sharing features [37]. A meta-analysis examining the provision of mental health services through mobile apps for adolescents highlighted the positive effects of these apps on psychological disorders, psychosocial strategies, and skills, as well as health-related symptoms and behaviors [38]. Furthermore, the analysis found variations in app use based on the users’ motivations for choosing specific apps, yet confirmed that all apps were effective, regardless of functions such as personalized services, gamification, notifications, or coaching [38]. An evaluation of the efficacy of online mental health promotion programs for adolescents indicated that these programs are generally effective in reducing subclinical symptoms of various mental disorders, while enhancing quality of life and psychological stability [39]. The forms of ICT used in these interventions included web platforms, mobile apps, SMS text messaging, virtual reality, and artificial intelligence chatbot tools. On the basis of the literature review, 17 service areas and 4 areas where expert consultation is preferred were identified and incorporated into the service needs assessment survey.

#### Smart Health Care Service Needs Assessment and Core UI Design

The findings from the needs assessment survey on smart health care services are presented in Table 1. The results showed that 83% (30/36) of the students and 97% (29/30) of the parents expressed a desire to participate in the implementation of smart health care services. In addition, 97% (35/36) of the students, 97% (29/30) of the parents, and 100% (30/30) of the health teachers considered the use of wearable devices in health care services to be beneficial. Smartphones were the most preferred devices for accessing smart health care services, receiving highest ratings from both students (mean 4.50, SD 0.65) and parents (mean 4.47, SD 0.57). Health teachers rated smartphones and wearable devices equally highly, with mean scores of 4.53 (SD 0.68) and 4.53 (SD 0.78), respectively. Regarding preferred areas of participation in health care services, students showed a strong preference for stress management (mean 4.44, SD 0.84), parents favored body shape management (mean 4.57, SD 0.68), and health teachers preferred expert individual prescriptions (mean 4.80, SD 0.48). Digital health readiness scores were 4.15 (SD 0.56) for students, 4.43 (SD 0.48) for parents, and 4.61 (SD 0.39) for health teachers. In terms of digital readiness characteristics, all 3 groups indicated that the use of digital devices was the most common method for obtaining health management and information. Smartphones were identified as the most familiar mobile devices across all groups. Notably, most participants in each group expressed reluctance to spend money on mobile health care services or the use of mobile devices. Finally, self-efficacy in using digital media systems was highest among health teachers (mean 4.53, SD 0.56), followed by middle school students (mean 4.34, SD 0.77) and parents (mean 4.22, SD 0.68).

**Table 1:** General characteristics of participants and characteristics related to smart health care services^a^.

Characteristics and categories				Student (n=36)		Parents (n=30)		School health teacher (n=30)
**Age group (years), n (%)**								
	20-29		—^b^		—		6 (20)	
	30-39		—		8 (27)		9 (30)	
	40-49		—		20 (67)		8 (27)	
	50-59		—		2 (7)		7 (23)	
**Sex, n (%)**								
	Male		11 (35)		5 (17)		—	
	Female		20 (65)		25 (83)		30 (100)	
**Willingness to participate in the service, n (%)**								
	Yes		30 (83)		29 (97)		—	
	No		6 (17)		1 (3)		—	
**Usefulness of wearable devices in the service, n (%)**								
	Usefulness		35 (97)		29 (97)		30 (100)	
	Unusefulness		1 (3)		1 (3)		—	
**Service device preferences (out of 5 points), mean (SD)**								
	Smartphone		4.50 (0.65)		4.47 (0.57)		4.53 (0.68)	
	Wearable devices		3.94 (0.95)		4.30 (0.70)		4.53 (0.78)	
	Computer		3.19 (1.09)		3.17 (1.05)		3.37 (0.56)	
	Tablet PC		3.67 (0.99)		3.60 (0.89)		3.70 (0.79)	
	Television		2.83 (1.08)		3.17 (0.79)		3.00 (0.64)	
**Participation preference by service area, mean (SD)**								
	Physical activity		4.28 (0.88)		4.37 (0.61)		4.67 (0.55)	
	Nutrition		4.42 (0.65)		4.33 (0.61)		4.53 (0.63)	
	Obesity prevention and management		4.17 (0.74)		4.23 (0.97)		4.40 (0.62)	
	Body shape management		4.42 (0.69)		4.57 (0.68)		4.40 (0.67)	
	Mental health		4.19 (0.89)		4.37 (0.76)		4.13 (0.82)	
	Disease prevention and management		4.00 (0.93)		4.17 (0.70)		4.00 (0.83)	
	Drug abuse		3.83 (1.08)		3.87 (1.07)		3.93 (1.11)	
	Smoking and drinking		3.61 (1.25)		3.57 (1.28)		3.70 (1.18)	
	Stress management		4.44 (0.84)		4.50 (0.68)		4.27 (0.98)	
	Sex education		3.72 (1.16)		4.10 (0.84)		3.97 (0.96)	
	Addiction of computer and smartphone		3.94 (1.09)		4.30 (0.79)		3.73 (1.11)	
	Sleep		4.31 (0.82)		4.53 (0.68)		4.23 (1.01)	
	Healthy living skills		4.11 (0.92)		4.53 (0.63)		4.10 (1.18)	
	Life safety		3.92 (0.94)		3.97 (1.13)		3.63 (1.07)	
	First aid		4.28 (0.94)		4.47 (0.63)		4.43 (0.77)	
	Expert consultation		3.94 (1.09)		4.10 (0.92)		4.67 (0.61)	
	Expert individual prescription		3.92 (1.05)		4.23 (0.77)		4.80 (0.48)	
**Preferred areas for expert consultation and assessment, mean (SD)**								
	Physical activity		4.42 (0.77)		4.37 (0.76)		4.67 (0.55)	
	Nutrition		4.28 (0.70)		4.20 (0.76)		4.53 (0.68)	
	Obesity prevention and management		4.25 (0.77)		4.23 (0.90)		4.40 (0.77)	
	Body shape management		4.42 (0.69)		4.53 (0.73)		4.47 (0.78)	
**Digital health readiness, mean (SD)**								
	Total		4.15 (0.56)		4.43 (0.48)		4.61 (0.39)	
	Mobile services capability		4.29 (0.59)		4.50 (0.53)		4.54 (0.54)	
	Perception of the importance of mHealth^c^ apps and devices		4.24 (0.68)		4.30 (0.66)		4.49 (0.57)	
	mHealth literacy		4.09 (0.67)		4.46 (0.51)		4.69 (0.44)	
	Health equity		4.28 (0.68)		4.47 (0.64)		4.71 (0.38)	
**Digital readiness, n (%)**								
	**Methods of acquiring health information and managing health^d^**							
		Digital device	27 (87)		24 (80)		24 (80)	
		Computer	5 (16)		10 (33)		20 (67)	
		Health care professional	4 (13)		13 (43)		13 (43)	
		Social relationships	8 (26)		7 (23)		1 (3)	
		Print media	2 (7)		5 (17)		11 (37)	
		Broadcasting media	1 (3)		5 (17)		5 (17)	
	**Familiar mobile devices^d^**							
		Smartphone	29 (93)		29 (97)		29 (97)	
		Tablet PC	7 (23)		8 (27)		18 (60)	
		Wearable devices	9 (29)		13 (43)		24 (80)	
		Mobile medical devices	1 (3)		4 (13)		17 (57)	
	**Digital health use experience^d^**							
		Health care app	20 (65)		25 (83)		24 (80)	
		Take a training course	7 (23)		6 (20)		14 (47)	
		Participate in health care program	5 (16)		4 (13)		5 (17)	
		Video consultation or telemedicine	2 (7)		1 (3)		4 (13)	
		Others	3 (10)		2 (7)		1 (3)	
**Willingness to pay for mobile health care services (KRW), n (%)**								
	No intention to pay		22 (71)		12 (40)		15 (50)	
	1000 (US $0.7) per month		3 (10)		10 (33)		10 (33)	
	5000 (US $3.6) per month		5 (16)		5 (17)		3 (10)	
	10,000 (US $7.2) per month		1 (3)		3 (10)		2 (7)	
**Willingness to pay for mobile device purchase (KRW), n (%)**								
	No intention to pay		23 (74)		14 (47)		10 (33)	
	<10,000 (US $7.2)		4 (13)		8 (27)		4 (13)	
	<50,000 (US $36.2)		2 (7)		5 (17)		7 (23)	
	<100,000 (US $72.4)		1 (3)		3 (10)		9 (30)	
	≥100,000 (US $72.4)		1 (3)		—		—	
Self-efficacy using digital media systems, mean (SD)				4.34 (0.77)		4.22 (0.68)		4.53 (0.56)

^a^This table presents the results of general characteristics and those related to mobile health care services (digital readiness and self-efficacy in using digital media systems) gathered from research participants via a web survey of 47 questions. The survey recruited 36 first-year students, 30 parents from 4 middle schools in Seoul, and 30 health teachers employed at middle schools in Seoul to develop a smart health management service for adolescents in South Korea. The web survey was conducted between December 3 and 23, 2023.

^b^Not available.

^c^mHealth: mobile health.

^d^Multiple responses.

The primary persona centers on middle school students, the main target audience for this service, while the subpersonas include parents, teachers, and school health educators who support these students in engaging with the service. In addition, the research team presented a previously developed child health management app to solicit feedback on the core UI. During the interviews, parents and school health teachers indicated that effective adolescent health care is often considered more critical than the students recognize. The interview results are shown in Table 2. All 3 groups—students, parents, and health teachers—consistently identified diet and exercise as the key focus areas for managing adolescent health. Moreover, school health teachers emphasized the importance of addressing smartphone use, sleep patterns, health management motivation, caffeine addiction, and mental health as vital components of adolescent well-being. Students recognized the importance of exercise and diet management but struggled to implement these practices independently. Parents consistently tried to oversee their children's dietary and exercise routines; however, they often experienced stress due to the challenges of managing these aspects of their children's health. In addition, adolescents lack sufficient leisure time and deal with challenges caused by the influence of the media on eating habits. School health teachers attempted to address students’ health issues by providing health management information during class and offering individual counseling or expert consultations when needed. However, they encountered difficulties due to students’ academic pressures and the lack of support at home. Students expressed a desire for an app that would allow them to engage with friends. At the same time, parents sought an app offering simple diet recommendations and a function to interpret data from wearable devices. School health teachers aimed for an app that could track sleep, eating habits, and exercise amounts in 1 place and facilitate friendship building to enhance students’ social skills. To tackle the key health challenges facing adolescents, all 3 groups emphasized the necessity for functions that evaluate and restrict computer or smartphone use. Furthermore, student and parent groups highlighted the importance of providing incentives for continued service use.

**Table 2:** Interview findings to comprehend user needs and collect feedback on core user interface (UI)^a^.

Categories			Student		Parents		School health teacher
**Health care**							
	The importance of health care for adolescents (out of 10), mean (SD)	7.05 (1.77)		9.13 (1.04)		9.10 (1.22)	
	The most essential thing in adolescent health care	Diet management and exercise		Diet management and exercise		Diet management, exercise, smartphone use, sleep habits, body image, health care motivation, caffeine addiction, and mental health	
	Efforts to take care of your health (efforts to take care of your children’s or students’ health)	Most students do not exercise enough They know about diet management, but they do not practice it		Many parents continue to make efforts to manage their children’s diet and exercise		Providing health management information using health class time, checking one’s problems, individual counseling for students with issues, and providing counseling when necessary	
	Anything that interferes with or makes it difficult to take care of your health	Many students say that exercise is a hassle It is not easy to control your diet		Controlling children causes stress and conflict Children do not eat, and there is no time due to school, etc A lot of exposure to food consumption videos on YouTube, etc Parents also find it annoying and difficult to practice consistently		Smartphone use Academic burden Lack of supporters at home Friends Increased PC use due to remote classes	
	The app you want for your health care	Apps you can use with friends		An app that explains simple exercises An app that allows users to view InBody results alongside exercise data, provides simple diet recommendations, and includes a feature to interpret data from wearable devices		An app that lets you see your sleep, eating habits, and exercise all at once An app that warns you when you do not move for a long time An app that lets you make friends to improve your social skills	
	What you need to know about adolescent health care from the smart health care service (personas)	Creating life rules, managing diet, evaluating smartphone use and limiting use time, recommending exercise, providing visual stimulation by reflecting avatars in case of underweight, introducing friends, counseling, etc Desiring tangible rewards for consistent effort		Mental health support, activity assessment, computer and smartphone use assessment, and use time limit function, easy meal preparation, exercise method, anonymous counseling, and eating habit formation Desiring tangible rewards for consistent effort		Healthy body image education, diet plan, anonymous counseling and chat room, diet diary, and social networking site use time evaluation Desiring tangible rewards for consistent effort	
**Comments on the core UI of the app**							
	Convenience or interest of the App	Characters are childish Easy to upload food photos		Characters are cute compared to the target age group Simple and conveniently configured Students probably will not evaluate their diet every time		Good ranking and meal photo upload function Can be used when counseling students Characters are childish It would be good if there were rewards	
	Reason for using the app	—^b^		The fact that students can participate on their own, the diet evaluation function, and the health coach feedback function are good I want to use it to form regular living habits I want to use it because it allows for objective evaluation of my child’s weight		When checking the student health record, it is not effortless, so it is convenient to use the app to check quickly It would be good to give gifts through rankings for each class during health classes Easy for students to manage themselves and motivate them Systematic management is possible when participating in the school health care program Use as a means to manage students who lack management at home	
	The part of the app you want to add	Added desired functions: goal setting, adding friends, chatting, and character (theme) change Bonuses are provided when goals are met Many participants preferred team play over individual participation and said they wanted to choose a participation method based on their personal preferences		Weight loss can be observed in cases of obesity. Still, as weight gain is average for adolescents, it would be better if the evaluation was centered on BMI and the result evaluation function included evaluation of weight and muscle mass, etc A function that recommends a favorite exercise reflecting the user’s preference A function to change characters A function to send a message to a health coach The scope of information sharing desired in the parent app was different Many participants prefer team play over individual play, and it is hoped that this can be changed depending on preference		Want to add mental health-related content It would be good to input weight linked to a scale and to let me know my current location when entering height, weight, and age in addition to weight If BMI is low, a warning message is displayed when weight is decreasing If a diet photo is viewed and there is a lot of spicy food (yeop-tteok, mala-tang, etc), a warning message is displayed for provocative diets Character change Function to share diet photos, etc Function for health teachers to send feedback to students Evaluation of mobile phone use time They want to provide a way for young people to choose between individual or team participation	
	Frequency of use or effect of use when using the app (when school health teachers provide a web for teachers)	The desired frequency of use varies from daily to once a week It will be effective if used in the long term		Many say that it is difficult to use every day and can be used a few times a week in the evening It is said that it will be used for exercise or health management review purposes		Observation of health care outcomes -Counseling and guidance for students with problems Desire to provide necessary information for health teachers in addition to student-related information Use during school health care programs	
	Expected problems when using the app	They will not use it because it is annoying Need motivation or rewards for continued use Difficulties due to smartphone data use restrictions		It may be difficult for teenagers to continue using the app		Ongoing guidance will be necessary to encourage continued app use by users Motivation to use the app is needed	

^a^This table displays the results of interviews with individuals who participated in the needs assessment and core UI design of the adolescent smart health management service. A total of 30 participants took part in the interviews, which lasted approximately 20 minutes each. The participants included 30 volunteers (10 students, 10 parents, and 10 school health teachers) out of 96 respondents in the survey for this study. The interviews were conducted from December 3 to 23, 2023, through either face-to-face meetings or online videoconferencing. The interviews used 11 semistructured questions, organized according to the categories shown in this table.

^b^Not available.

Following a review of feedback on the UI for the adolescent health management app, it was found that 8 (80%) out of 10 students expressed a desire to use the app. Students indicated that if the app included multiple features, clear explanations would be essential; and they preferred to use it collaboratively with friends. Desired additional functions included goal setting, the ability to add friends, chat features, and character customization (themes). Parents appreciated that their children could actively engage with the app and evaluated the diet assessment and health coach feedback functions positively. However, they noted that maintaining long-term use of the app might be more challenging than its initial use. They suggested that a BMI evaluation function would be more beneficial than simply recording weight, given the growth characteristics of adolescent. In addition, the health teachers also praised the ranking system, the diet photo upload feature, and the app’s capability to quickly convey health management status. They expressed interest in including features related to mental health, warning notifications for concerning weight loss, alerts for inappropriate diet photos, and a feedback mechanism for communicating with health teachers. Both students and parents showed a preference for team-oriented participation over individual engagement when using the health management app. All 3 group expressed the desire for an option that would allow them to choose between individual and team play based on their participation preferences. On the basis of the insights gathered from literature reviews, surveys, and interviews, a comprehensive list of user needs for the app was developed, leading to a refined core UI design that reflects these requirements.

#### Usability Evaluation of Smart Health Care Service Core UI

The usability evaluation of the core UI assessed the significance, service preference, and potential for the main service areas (sleep, weight, diet management, physical activity measurement, smartphone use assessment, and health points [rewards]) identified through literature review and needs assessment (Table 3). A heuristic and usability evaluation of the app using the developed core UI was conducted. The findings from the importance evaluation indicated that students and parents deemed the sleep function as the most critical, while health teachers prioritized physical activity measurement. Regarding service preferences, students and parents showed a strong preference for sleep and weight management, whereas health teachers favored physical activity measurement and diet management. Both parents and health teachers believed that physical activity measurement had the most significant potential to address health-related issues. At the same time, students felt that providing health points (rewards) was the most effective solution. The heuristic evaluation of the core UI revealed a severity level ranging from slight to moderate, with students scoring an average of 1.65 (SD 0.47), parents an average of 1.38 (SD 0.47), and school nurses an average of 1.15 (SD 0.27). Regarding usability evaluation of the core UI, school nurses rated its effectiveness and usefulness highest, scoring 4.26 (SD 0.41) and 4.18 (SD 0.54), respectively. In addition, school health teachers rated usability and user control most favorably, with scores of 4.29 (SD 0.55) and 4.08 (SD 0.60), respectively.

**Table 3:** The result of the usability evaluation of core user interface (UI)a.

Characteristics and categories			Student (n=23), mean (SD)	Parents (n=24), mean (SD)		School health teacher (n=29), mean (SD)
**Importance of core UI**						
	Sleep management	3.83 (0.49)		3.67 (0.48)	3.59 (0.73)	
	Weight management	3.52 (0.59)		3.33 (0.48)	3.41 (0.73)	
	Diet management	3.52 (0.59)		3.42 (0.50)	3.66 (0.48)	
	Physical activity measurement	3.35 (0.71)		3.33 (0.48)	3.79 (0.41)	
	Smartphone use assessment	3.30 (0.70)		3.33 (0.56)	3.10 (0.82)	
	Health points award (reward)	3.30 (0.70)		3.25 (0.44)	3.45 (0.57)	
**Service preference**						
	Sleep management	3.87 (0.34)		3.58 (0.50)	3.45 (0.69)	
	Weight management	3.65 (0.49)		3.46 (0.51)	3.52 (0.69)	
	Diet management	3.52 (0.59)		3.29 (0.55)	3.66 (0.48)	
	Physical activity measurement	3.17 (0.89)		3.33 (0.56)	3.72 (0.45)	
	Smartphone use assessment	3.13 (0.92)		3.33 (0.56)	3.00 (0.80)	
	Health points award (reward)	3.43 (0.73)		3.33 (0.48)	3.45 (0.69)	
**Possibility of solving health problems**						
	Sleep management	3.52 (0.67)		3.29 (0.62)	3.17 (0.60)	
	Weight management	3.35 (0.57)		3.21 (0.51)	3.38 (0.56)	
	Diet management	3.43 (0.59)		3.13 (0.54)	3.21 (0.68)	
	Physical activity measurement	3.48 (0.59)		3.42 (0.50)	3.76 (0.51)	
	Smartphone use assessment	3.48 (0.67)		3.33 (0.48)	3.59 (0.57)	
	Health points award (reward)	3.61 (0.58)		3.21 (0.51)	3.52 (0.57)	
**Heuristic evaluation of core UI^b^**						
	Total	1.65 (0.47)		1.38 (0.47)	1.15 (0.27)	
	Immediate notification	1.81 (0.66)		1.45 (0.60)	1.28 (0.46)	
	Familiar rules	1.73 (0.70)		1.36 (0.49)	1.16 (0.37)	
	Prevent problems	1.67 (0.62)		1.36 (0.49)	1.11 (0.32)	
	Display	1.80 (0.68)		1.24 (0.44)	1.15 (0.46)	
	Concentration	1.73 (0.70)		1.32 (0.48)	1.16 (0.37)	
	Visually pleasing interface	1.50 (0.65)		1.33 (0.48)	1.13 (0.34)	
	Intuitive interfaces	1.73 (0.80)		1.43 (0.66)	1.12 (0.33)	
	Clear navigable path	1.60 (0.51)		1.35 (0.59)	1.08 (0.28)	
	Allow shortcut	1.57 (0.65)		1.43 (0.60)	1.15 (0.36)	
	Support for various environments	1.50 (0.52)		1.33 (0.48)	1.12 (0.33)	
	Easier input	1.57 (0.76)		1.41 (0.59)	1.08 (0.28)	
	Use camera	1.56 (0.73)		1.36 (0.49)	1.12 (0.33)	
**Usability evaluation of core UI**						
	Impact	4.13 (0.88)		4.17 (0.61)	4.26 (0.41)	
	Perceived usefulness	4.08 (0.71)		4.12 (0.58)	4.18 (0.54)	
	Perceived ease of use	4.11 (0.71)		4.03 (0.51)	4.29 (0.55)	
	User control	4.03 (0.68)		4.04 (0.74)	4.08 (0.60)	

^a^This usability evaluation results from an assessment of the core UI developed based on findings from a needs assessment and feedback collected about the core UI. The participants in the usability evaluation included 23 first-year students, 24 parents from 4 middle schools in Seoul, and 29 school health teachers from middle schools in Seoul. The evaluation comprised a total of 50 questions: 6 concerning the importance of UI elements, 6 relating to potential problem resolution, 6 measuring user preference, 12 aimed at the heuristic evaluation of mobile apps, and 20 focused on assessing the usability of the core UI. A web survey was conducted during the evaluation period from January 3 to 14, 2024.

^b^A total of 16 students responded.

### Development of MUZZIM

#### Development of User Flowchart for Apps and Web to Provide MUZZIM Service

Following the findings from the usability evaluation, the UI for both the app and website has been finalized, as detailed in Table 4. In the student app, a mission challenge feature was introduced to address feedback for team play, along with a selection button on the main screen that allows users to choose between individual or team play based on their preference. The diet management section offers evaluations based on photo uploads, while the weight management feature enables users to track their changes using BMI. An exchange center has also been incorporated, where users can earn health points within the app and redeem them for real-life products, such as convenience store gift certificates. A chatbot has been implemented to assist with system use, along with a button that provides direct access to the metaverse for engaging with health content. In the parent app, users can monitor their child’s overall health management status through a dashboard displaying the health coach’s report. In addition, the app includes an event mission feature, encouraging participation in activities with the child and the opportunity to earn points. The health teacher web interface features a dashboard that visualizes class achievement levels for shared challenges and a “Mission Challenge Participation” section that allows students to track their involvement in individual missions. The content is designed to facilitate health monitoring for each item and provides health management progress statistics by class. Moreover, a feedback mechanism has been implemented to review health coaches, expert feedback, and consultation outcomes, enabling communication of this feedback to parents or students. A user flowchart was developed based on the UI design to illustrate specific use paths for users of both the app and web services.

**Table 4:** Contents of the mobile app (students and parents) and web (health teachers)^a^.

Categories, subcategories, and mobile app content				Description
**App for student**				
	**Diet management**			
		Upload a photo	Students upload photos of their meals	
		Diet evaluation	Diet evaluation through photo analysis	
		Write a food diary	Write your thoughts about the meal, etc	
	**Weight management**			
		Enter your height and weight	Entered directly by the student	
		BMI graph	Displays the normal BMI range along with my current status within that range	
	Sleep management	Visualize your sleep records	Visualize total sleep time, actual sleep time, and wake-up time during sleep measured by a smartwatch, and calculate sleep efficiency through this	
	**Smartphone use assessment**			
		Visualization of smartphone use time	Provides visual information on target use time and actual use time per day	
		Set use time	First, set a target time for smartphone use	
		Visualize app use time	Provides visual information on the cumulative use time of apps used throughout the day	
	**Mission challenge**			
		Common mission	10,000 steps challenge	
		Diet, sleep, and mental health	You can participate in the challenge by selecting from the 3 missions presented. The missions are presented in 3 stages for each area. It is a form of performing the given missions while competing with individuals or teams	
	**Health point**			
		Current points	Present current points and next point payment date	
		Point exchange	Exchange points for actual products (convenience store gift certificates, etc)	
	**Physical activity measurement**			
		Step count	The number of steps measured by the smartwatch, the calories burned calculated from this, and the total distance traveled are presented	
		Heart rate	Heart rate measured by smartwatch, daily average heart rate	
		Stress	Visualize and present the stress levels calculated from heart rate measured by a smartwatch (including related interpretation), and present the average daily stress values for the past 5 days in a graph	
	Chatbot	N/A^b^	The top of the main screen displays a toggle button that allows you to move to the Kakao Chatbot	
	Metaverse	N/A	The top of the main screen features a toggle button that allows you to move to the metaverse platform	
**App for parents**				
	Dashboard	N/A	Provides visualization of your child’s mission challenge participation status (selection steps and level of achievement for each area)	
	Health coach report	N/A	Receive health information, such as your student’s health status, and simple recipe recommendations from a health coach every week	
	Challenge missions with students	N/A	Choose and complete a mission with your children	
	Event mission	N/A	Complete missions that allow you to obtain health points other than mission challenges	
**Web for health teacher**				
	Dashboard	N/A	Visualization of the level of achievement and ranking of the common challenge for each class	
	Participate in a mission challenge	Walking, diet, sleep, and mental health	Check individual participation status in each area	
	Health monitoring	Diet, weight, sleep, physical activity, smartphone use, health point, chatbot, and metaverse	Provides comprehensive information on monitoring results for each item	
	Progress of health management by class	N/A	Check health management progress statistics by class	
	Feedback	Health coach report status, teacher report confirmation, message sending to students, expert request status and results, consultation results, and parent message sending function	Check information about feedback within health care services from health coaches, teachers, students, professionals, parents, etc	

^a^A comprehensive list of the app’s and website’s user interface contents based on the results of a usability evaluation regarding the demand for adolescent smart health management services and essential user interfaces.

^b^N/A: not applicable.

#### Algorithm Development of MUZZIM and Design of Lifelog Data Collection System

**Figure 2:**
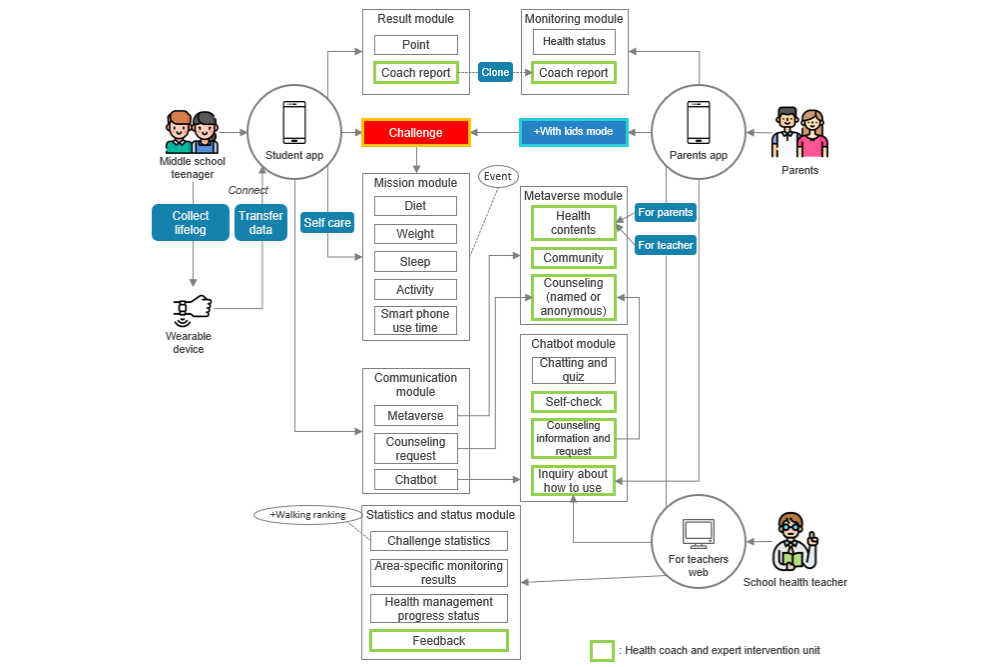
Use case diagram of the smart health care service.

A use case diagram has been developed to illustrate the interactions between users of this service and the various devices involved, including mobile apps, web interfaces, wearable devices, a metaverse platform, and chatbots (Figure 2).

**Figure 3:**
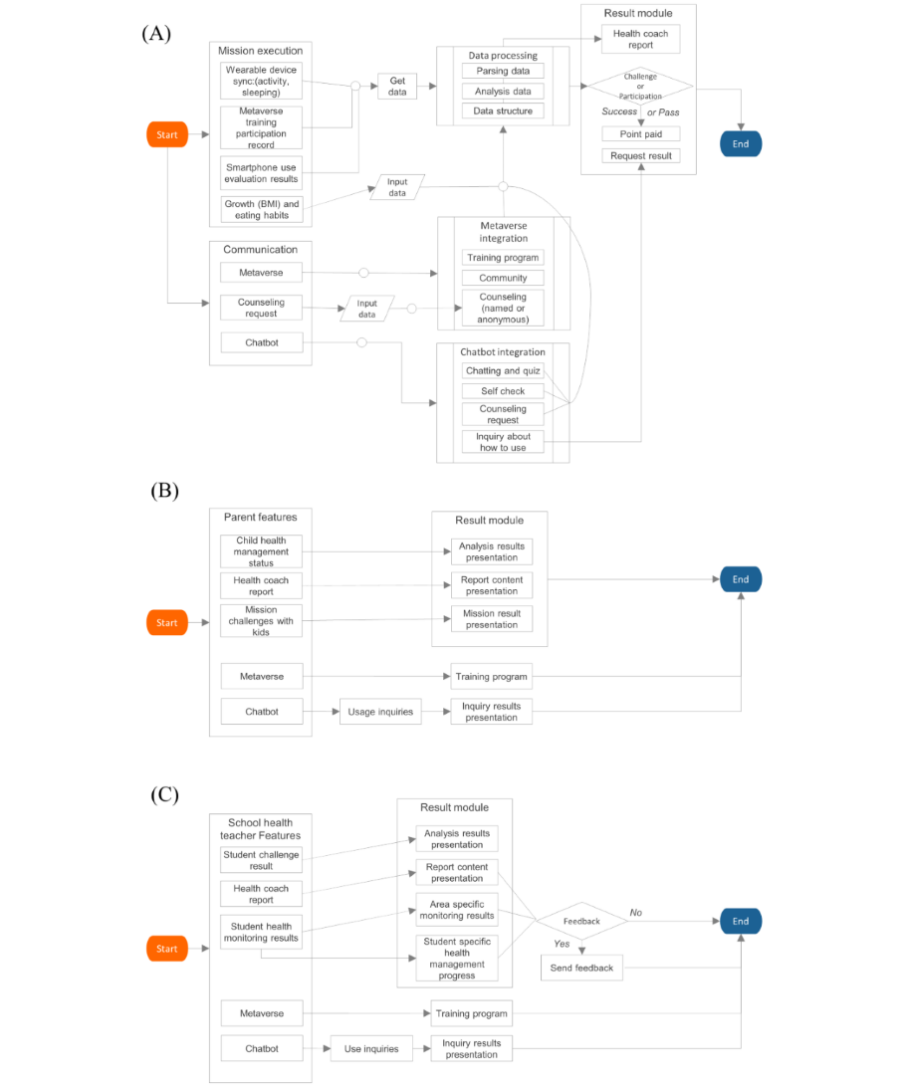
Flowchart for visualizing MUZZIM service algorithms for (A) students, (B) parents, and (C) school health teachers.

**Figure 4:**
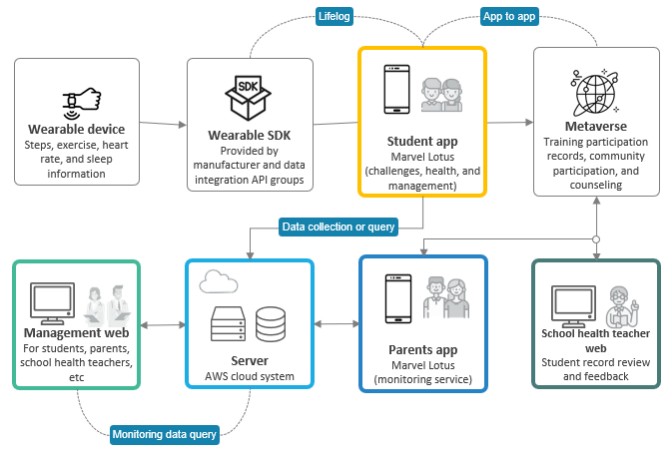
Lifelog data collection system for the MUZZIM service. API: application programming interface; AWS: Amazon Web Services.

This service primarily focuses on health care by leveraging apps for students and parents, alongside web access for health teachers. Students engage in challenge missions designed to enhance self-management through the app, while lifelog data—such as activity and sleep metrics—gathered by a wearable device, specifically a smartwatch, are transmitted to the app. Users can access the metaverse and chatbot features within the app’s communication module, enabling them to request consultations when necessary. On the basis of mission performance and the data collected, users earn points and receive reports from health coaches in the results module. The metaverse module offers health promotion content, facilitates both real-name and anonymous consultations, and includes community features for student interaction. The chatbot module supports disease self-diagnosis, consultation information provision, consultation requests, and service use guidance. In the parent app, the monitoring module enables parents to view their child’s health information and the health coach report received. The web interface for health teachers provides statistical data on mission challenges and area monitoring within the statistics and status module, allowing teachers to identify students needing feedback and provide the necessary guidance. A flowchart visualizing the algorithm of this service is depicted in Figure 3, while Figure 4 presents a schematic diagram illustrating the collection system of the selected smartband and the lifelog data gathered during service use.

## Discussion

### Principal Findings

This study aims to design “MUZZIM,” a smart health care service dedicated to promoting the health of adolescents. We developed the core UI for both the app and web services through a comprehensive literature review and a needs assessment survey. “MUZZIM” specifically targets first-year middle school students, with parents and health teachers as key supporters in managing student health. The MUZZIM service uses various platforms, including apps for students and parents, a web portal for health teachers, wearable devices (smartwatch), metaverse platforms, and chatbots, to enhance service delivery. To design the core UI, we examined 65 existing health care services and conducted a user needs assessment survey involving 96 participants and in-depth interviews with 30 individuals. The insights gained from the literature review and user needs assessment informed the design of the core UI (web and app). A usability evaluation conducted with 76 participants further refined this design. Consequently, we created a flowchart visualizing the user journey through the app and web within the MUZZIM service, alongside a service provision algorithm. In addition, we developed a schematic diagram outlining the collection system for lifelog data used in this service.

In this study, we developed the school-based MUZZIM service. Adolescents spend about one-third of their day in school, which makes school-based health care services particularly effective [40]. In addition, these services offer comprehensive support to all students, making them convenient and accessible for adolescents considered vulnerable, thereby helping to minimize health disparities among this age group [41]. According to Rungan et al [42], school-based health services have been shown to enhance treatment accessibility, improve health and educational outcomes, provide high satisfaction levels for students and parents, and demonstrate cost-effectiveness. Furthermore, the current use of health checkup results has been inadequate, despite requirements outlined in domestic school health laws. Therefore, implementing these services within schools is deemed appropriate to leverage health checkup outcomes actively and monitor future progress and changes. In this study, we plan to introduce a web page that allows school health teachers to participate as users, facilitating effective monitoring and feedback for improved health management.

Adolescents possess distinct characteristics that differentiate them from other age groups, primarily due to their developmental stage. They tend to prioritize friendships over familial relationships and strongly desire autonomy in their lives [9]. Consequently, grasping adolescents’ perspectives is vital for effectively designing and enhancing health and social services [43]. To ensure that these services meet adolescents’ needs, this study conducted a comprehensive literature review, identifying the needs of both adolescents and their parents and input from school health teachers regarding the design of a core UI. Furthermore, the UI was evaluated for usability to ensure its effectiveness. The literature review involved analyzing previous studies and reports, both domestically and internationally, to gather data on ICT health management service areas tailored for children and adolescents, areas for expert consultation, and strategies to encourage service participation. The review identified 17 health care service provision areas and 4 expert consultation areas deemed appropriate for this service. It was noted that most health management apps offered training coaching or voice coaching to encourage physical activity [32]. Furthermore, it was confirmed that strategies such as providing feedback, offering integrated content, incorporating engaging elements, fostering motivation, creating an environment conducive to physical activity, and including gamification were used to ensure ongoing use of the health management service [33]. In diet management, a clear need for parental involvement, food diary tracking and monitoring, personalized diet plans, diet evaluation features, and tailored content based on performance and age was established [35,44]. Positive feedback, recognition, rewards, and notification functionalities emerged as key motivators for using diet management apps, with notable differences observed between genders [45].

The survey results aimed at identifying user needs revealed that most students (30/36, 83%) and parents (29/30, 97%) expressed interest in participating in the smart health care service. Among all 3 groups surveyed, the predominant method for obtaining health information was through digital devices, with smartphones being the most commonly used. In addition, most respondents indicated a preference for receiving services via smartphones. Furthermore, an impressive 97% (35/36) of the students, 97% (29/30) of the parents, and 100% (30/30) of the health teachers found using wearable devices for service provision beneficial. This indicates a high rate of smartphone use and digital familiarity among the youth in our country [46]. However, even if it is a familiar form of service, it is necessary to confirm in future research whether there are practical barriers to its use and whether its use can bring about improvements in health outcomes. The findings also revealed that students showed a strong demand for stress management within the health management services, corresponding with the global prevalence of psychological stress among adolescents aged 10 to 19 years, which stands at approximately 25% [47]. This aligns with the fact that 36.4% of domestic middle school students reported being aware of their stress levels, and mental health counseling was identified as a primary concern, being the highest at 24.1% [48]. In addition, parents exhibited a keen interest in body management, while health teachers favored tailored prescriptions from experts.

The assessment of digital health readiness revealed that mHealth literacy scores averaged 4.09 (SD 0.67) for students, 4.46 (SD 0.51) for parents, and 4.69 (SD 0.44) for health teachers. A low level of mHealth literacy complicates the ability to locate and comprehend digital health information, ultimately hindering the effective use of digital health care services [49]. Among these groups, students exhibited a notably lower level of mHealth literacy, indicating a clear need for targeted support in using these services. Furthermore, Sharma et al [50] mentioned that young people may lack knowledge about the use of digital technology. Chatbots have the potential to make health care more accessible to people, providing quick responses to inquiries and eliminating the need for lengthy navigation to obtain specific information [51]. In addition, the effectiveness of chatbots has been proven in various health care services [52-55]. Therefore, chatbot services were introduced to help with service use methods, etc. The chatbot will be composed of frequently asked questions regarding service use guides, device operation methods, and service schedules, available every time.

Most participants in the needs assessment survey expressed an unwillingness to pay for mobile health care services or devices. Aydin and Silahtaroglu [56] and Wang and Qi [57] also noted that a considerable number of individuals refrain from using health care apps due to their costs, highlighting that financial constraints are a significant barrier to accessing mobile health care services. Jacob et al [58] emphasized that the direct costs of app use and additional expenses related to data consumption can deter users. Sharma et al [50] cited that some youth have limited access to high-speed internet or digital devices. Consequently, it appears more appropriate to provide health care services at no charge or, if necessary, adopt a policy framework where entities such as school health programs act as the purchasers rather than placing the financial burden on individuals. As part of the South Korean government’s digital health care initiative, the Korea Institute for Health Promotion and Development has been developing a public mobile health care platform since 2016 to provide digital health care services [59]. This service targets adults, children, adolescents, and older adults aged >65 years and strives to improve digital equity by providing customized digital health care services linked to wearable devices [59]. The Ministry of Education has also been trying to develop and introduce digital textbooks since 2007. They are also continuing their efforts to improve digital equity, such as planning to introduce an open learning platform and artificial intelligence textbooks [60].

In conducting interviews to understand user needs, personas and scenarios expressing recent health issues of adolescents were developed and used through expert advice. This was done based on the fact that qualitative data collection, such as interviews in the study by Graf et al [61], is limited to personal experiences and may have limitations in generalization. Interview results indicated that parents and health teachers perceived adolescent health management as more essential than the students did. This result is consistent with the result that 84.8% of middle school students in South Korea evaluated themselves as healthy [48], indicating that it is necessary to raise awareness of the need for health management at the primary prevention level, considering the characteristics of adolescence when lifelong health habits are formed. Students and parents reported that diet management and exercise are the most important aspects of adolescent health care, yet they are challenging to practice. This result is consistent with the reports of Agung et al [3], Eicher-Eicher-Miller et al [62], and Saleh et al [63] that although adolescents are aware of a healthy diet, various factors such as taste, cost, and availability of nutritious food options affect food selection and consumption. Therefore, it can be seen that to practice health behavior, it is necessary to consider various factors for practice beyond the level of simple knowledge transfer.

In exploring the desired functions for health care services, students preferred community features, reflecting the importance of peer groups within their age demographic. In contrast, parents indicated a need for capabilities that facilitate data collection from wearable devices, while health teachers sought an app that offers integrated information. In addition, health teachers noted the necessity for services addressing caffeine addiction alongside the areas identified through a comprehensive literature review. Adolescents are particularly susceptible to addiction to caffeine, and when they cease consumption, they may experience withdrawal symptoms such as anxiety, nervousness, headaches, and depression [64]. Notably, caffeine consumption among adolescents is on the rise; research by Oh [65] reveals that approximately 80% of adolescents have tried high-caffeine energy drinks, with 50.6% reporting side effects following consumption. Consequently, it is deemed essential to incorporate content related to caffeine consumption in the dietary education provided by MUZZIM.

To address the significant health issues faced by adolescents highlighted in the persona, all 3 groups emphasized the necessity of a feature to evaluate smartphone use. According to the 2022 Youth Statistics [48], 37% of adolescents in South Korea are at risk of smartphone overreliance, which reflects a clear understanding of the current situation. Students indicated a need for functions related to diet management and exercise recommendations, while parents expressed the importance of mental health support, activity level assessments, easy meal recipes, and anonymous counseling. Health teachers highlighted the need for education on healthy body image, anonymous counseling, a confidential chat space, and the use of food diaries. Furthermore, both students and parents stressed the importance of appropriate incentives for continued app use, aligning with the findings from the studies by Boushey et al [45] and Lee [66]. In addition, given the app’s various functions, it was deemed essential to provide guidance through a chatbot, tailored to the assessment of students’ mHealth literacy. Parents expressed a preference for evaluating BMI through both weight and height rather than solely inputting weight into the app. BMI serves as a crucial indicator for assessing an individual’s health status, demonstrating a strong correlation with various health conditions [67]. It is a cost-effective, straightforward, and safe metric suitable for large-scale adolescent populations, such as those in schools [68]. Given that adolescence is a period marked by rapid growth and development, weight fluctuations may occur that are not necessarily linked to health issues [69]. Therefore, for a service aimed at adolescents, monitoring BMI through the observation of changes in both weight and height is deemed more appropriate than focusing exclusively on weight. Furthermore, the heuristic evaluation of the app’s core UI was assessed as exhibiting slight to moderate severity levels. In terms of usability evaluation—encompassing effectiveness, usefulness, ease of use, and user control—the health teacher achieved the highest score, indicating strong expectations for using the app effectively. Heuristic evaluation is a method used to assess usability during the development phase of an app. It is important to conduct a reevaluation of usability once the app is implemented in actual adolescent health management. This reevaluation should involve collecting objective data, including metrics such as app retention rates and the frequency of use for various functions.

The MUZZIM service is designed to collect lifelog data using wearable devices to deliver customized services. It also facilitates feedback from health coaches and school health teachers regarding diet evaluations, BMI assessments, and overall health monitoring. Providing tailored services is crucial for enhancing the effectiveness of health care programs, particularly given the increasing demand for such personalized support [12]. To achieve this, activity data, including steps taken, heart rate, stress levels, and sleep duration, are gathered through wearable devices. In addition, height and weight measurements are manually entered to assess BMI. When students upload photos of their meals, the system analyzes these images and generates feedback, including warning messages about unbalanced diets based on input from school health teachers gathered during needs assessment. It is believed that these personalized service strategies will significantly contribute to meeting consumer preferences while also improving the effectiveness and overall satisfaction of the service.

The results of the literature review for the design of this study confirmed that gamification is effective in addition to rewards for continuous use, motivation, and increased effectiveness of health care services [34]. This is consistent with the results of Han et al [70] and Fleming et al [71]; when gamification was applied, the retention rate was high without increasing the risk to the participants compared to general treatment. Pramana et al [72] found that adding gamification elements to the mHealth service made it more valuable and appealing, resulting in higher use compared to the nongamified version. For this purpose, a mission challenge was included in the MUZZIM service. Common missions include a 10,000-step achievement challenge, and the diet, sleep, and mental health challenges were designed so that individuals can select and perform among the presented missions. In addition, reflecting the results of the needs assessment, the missions were designed so that individuals can perform them alone or in teams and compete according to their preferences. In addition, to create a class-by-class competition structure when providing the MUZZIM service in the future, a function was designed to check the scores of each class on the health teacher’s website. In addition, reflecting the opinion that rewards are necessary for continuous participation, health points were provided for health mission participation to encourage continuous participation. In addition, the current and upcoming point payment dates were provided, and a point exchange center was established where points could be redeemed for product vouchers, ensuring tangible rewards. These tangible rewards can help users develop more permanent habits [73]. In addition, as people tend to overvalue these tangible rewards, even small incentives can have a significant effect [74].

### Limitations

The limitations of this study are as follows. First, the service relies on various ICT technologies, which may restrict its accessibility based on the specifications of users’ smartphones and the availability of data. Consequently, when developing the service, it is essential to consider compatibility across different device models and the data use in users’ primary environments. Second, the service necessitates the involvement of a health coach who offers feedback throughout its operation. This structure also incurs ongoing operational costs due to the provision of rewards for service participation. Therefore, limited capital investment may pose challenges to sustaining the service. To reduce service costs, developing an algorithm that could replace feedback from health coaches might be beneficial. In addition, focusing on strategies that enhance self-efficacy in health management could promote sustainability, rather than relying solely on rewards for continuous service use. As smartphone dependency among adolescents has become a significant health concern, there may be challenges in the widespread adoption of this service due to potential resistance to digital platforms. Therefore, it is crucial to provide adequate explanations and feedback to raise awareness about the importance of the service. Finally, we made efforts to incorporate the needs of the service’s users—adolescents, parents, and health teachers—by conducting a survey. However, because the sample size was limited to 30 people and recruited from 1 city, the identified needs may not adequately represent the broader population. In addition, repeated surveys conducted on the same participants may raise reliability issues due to potential correlation. Therefore, future studies could reduce this concern by conducting sensitivity analysis or subgroup analysis. In addition, national statistical data, such as the Youth Health Behavior Survey conducted by the Korea Centers for Disease Control and Prevention, help to identify the needs of the target age group more effectively.

### Conclusions

This study aims to develop MUZZIM, a smart health care service tailored for adolescents. To design this service based on user needs, a literature review and a user needs assessment were conducted. Recognizing that adolescents are often digitally proficient, the latest ICT technologies have been incorporated into the service, including apps, websites, wearable devices, metaverse platforms, and chatbots. The specific operations of the MUZZIM service are illustrated through a user flowchart showcasing the UI, a use case diagram that highlights device interoperability, and additional flowcharts. Participating in health promotion services during adolescence can also affect future health by forming healthy habits. Therefore, it will be necessary to confirm whether the service is an initiative that can bring about positive health outcomes, such as improving adolescents’ health habits or health status, through an experimental study that provides the MUZZIM service designed in this study.

## Abbreviations

ICT: information and communication technology
MASUN: Method of App Selection based on User Needs
UI: user interface

## Data Availability

The datasets generated or analyzed during this study are available from the corresponding author on reasonable request.
